# The Metabolic Phenotype of Prostate Cancer

**DOI:** 10.3389/fonc.2017.00131

**Published:** 2017-06-19

**Authors:** Eric Eidelman, Jeffrey Twum-Ampofo, Jamal Ansari, Mohummad Minhaj Siddiqui

**Affiliations:** ^1^Division of Urology, Department of Surgery, University of Maryland School of Medicine, Baltimore MD, United States; ^2^The Veterans Health Administration Research and Development Service, Baltimore, MD, United States; ^3^Greenebaum Cancer Center, University of Maryland School of Medicine, Baltimore MD, United States

**Keywords:** prostatic neoplasms, metabolomics, metabolism, metabolic networks and pathways, precision medicine

## Abstract

Prostate cancer is the most common non-cutaneous cancer in men in the United States. Cancer metabolism has emerged as a contemporary topic of great interest for improved mechanistic understanding of tumorigenesis. Prostate cancer is a disease model of great interest from a metabolic perspective. Prostatic tissue exhibits unique metabolic activity under baseline conditions. Benign prostate cells accumulate zinc, and this excess zinc inhibits citrate oxidation and metabolism within the citric acid cycle, effectively resulting in citrate production. Malignant cells, however, actively oxidize citrate and resume more typical citric acid cycle function. Of further interest, prostate cancer does not exhibit the Warburg effect, an increase in glucose uptake, seen in many other cancers. These cellular metabolic differences and others are of clinical interest as they present a variety of potential therapeutic targets. Furthermore, understanding of the metabolic profile differences between benign prostate versus low- and high-grade prostate cancers also represents an avenue to better understand cancer progression and potentially develop new diagnostic testing. In this paper, we review the current state of knowledge on the metabolic phenotypes of prostate cancer.

## Introduction

Prostate cancer is one of the most commonly diagnosed cancers among men, particularly in the developed world. It represents not only a major factor in male morbidity and mortality but also an economic burden on the population (1, 2). Prostate cancer exhibits heterogenic properties among patients; however, there remain generalizable phenotypes associated with the majority of prostate cancers, including certain metabolic alterations (3, 4). With the rise of the field of study dedicated to cancer metabolomics, an effort has been made to evaluate prostate cancer through analysis of cellular metabolism in an effort to better understand the tumor pathogenesis as well as to create potential metabolically targeted treatments.

In addition to targeted treatments, the study of metabolism of cancer represents a new avenue for diagnostics (5). The tissue-specific activity of prostate epithelial cells has long been utilized in urologic oncology. A widely used prostate cancer marker is the prostate-specific antigen (PSA), which has been shown to be elevated in many men with prostatic disease (6). PSA testing is still used as an early screening method to determine the likelihood and progressive phenotype of prostate cancer. Recently, this method has undergone some scrutiny as it may lead to aggressive overtreatment in patients who may have been treated sufficiently with active surveillance (7). Improved tools for better risk stratification of prostate cancer patients are needed. This need emphasizes the importance of better understanding the metabolic mechanisms of prostate cancer (8). By better understanding the metabolism of prostate cancer, it may be possible to elucidate a metabolic biomarker whose levels may help to diagnose prostate cancer. This paper will discuss the current state of knowledge regarding prostate cancer metabolism and how it might relate to future treatment, screening, and diagnostic criteria.

## Normal Prostate Metabolism

**Figure 1:**
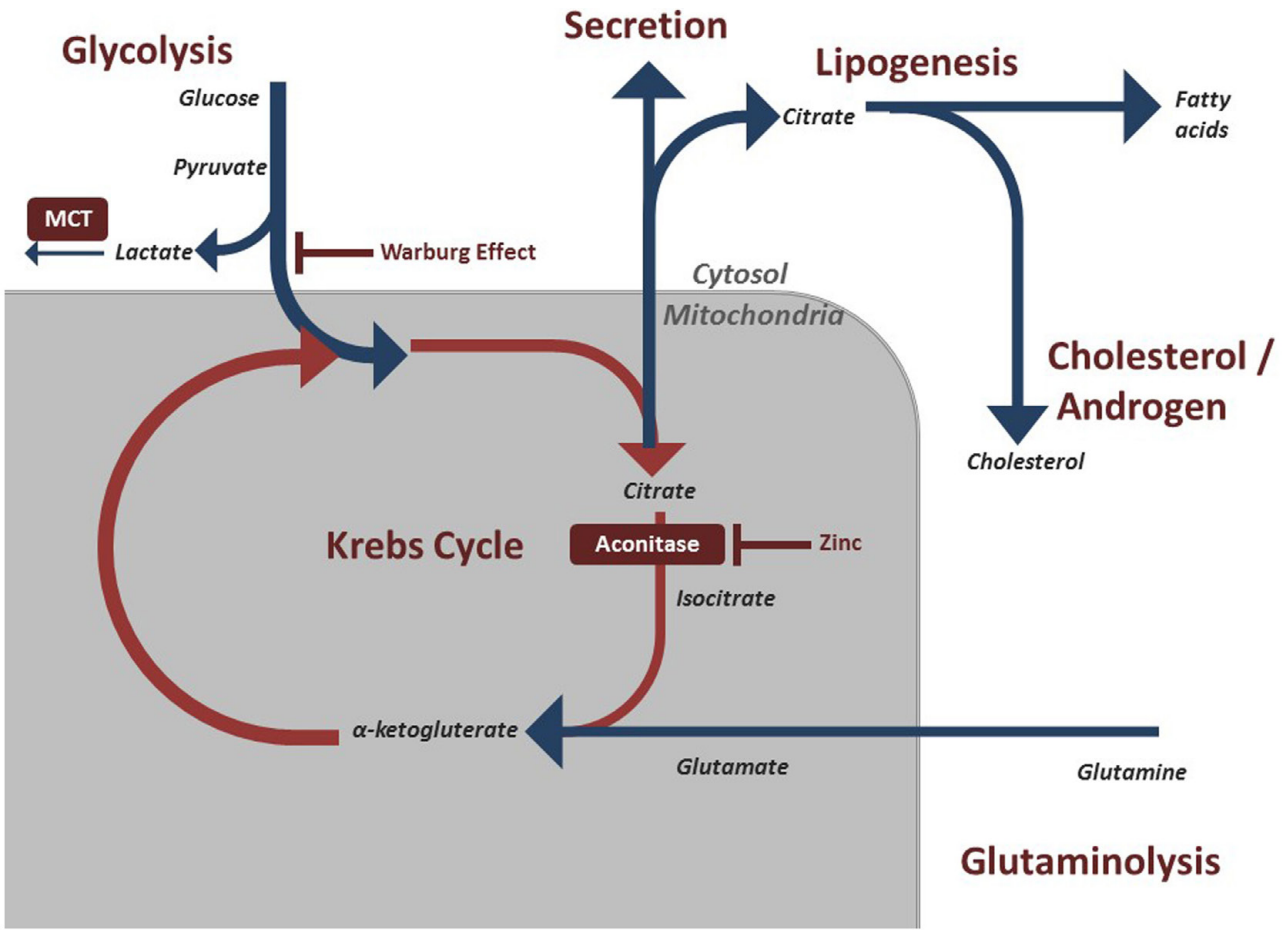
Regulated metabolic pathways involved in energy production and maintenance of prostatic cells. These pathways are often modified in malignant prostate cells to preserve cell division and growth.

In order to understand the metabolic alterations of prostate cancer, it is important to understand a key phenotype in benign prostate cells. Healthy prostate epithelial cells exhibit a highly specialized behavior regarding their metabolic pathways. Typically, cells rely on citrate oxidation as a key step in the Krebs cycle for the progression of aerobic respiration (9). However, prostate cells, notably the epithelial cells in the peripheral zone of the prostate are programmed to produce and not oxidize citrate (10). The citrate is subsequently secreted as a component of semen. The specialization of peripheral zone epithelium is of clinical interest as it is within this zone that prostate cancer begins (11). This unique process of citrate production is accomplished by another feature of prostate epithelial cells, the ability to accumulate large concentrations of zinc. Research on this pathway has indicated that high concentrations of zinc have an inhibitory effect on *m-aconitase*, the enzyme that catalyzes the oxidation of citrate within the Krebs cycle (Figure 1) (3). This zinc accumulation in benign prostatic epithelium is accomplished by increased amount of zinc transporter ZIP1 in this tissue (12). By accumulating citrate, normal prostate epithelial cells appear to halt the Krebs cycle and therefore act very different to the majority of cells in the body in the production of ATP.

## Warburg Effect

Metabolic modifications allow cancer cells to accommodate the massive energy requirement for rapid proliferation. Most solid tumors experience the Warburg effect, described in the early to mid 1900s by Otto Warburg, whereby malignant cells shift their dominant ATP producing pathway away from oxidative phosphorylation to aerobic glycolysis (13). This results in lactic acid fermentation (Figure 1) (14). This switch means that the cancerous cells display a significant increase in the rate of glucose uptake in order to meet the energetic need (13). Clinically, F-deoxyglucose positron emission tomography (FDG PET) scans take advantage of this trait as infused radiolabeled glucose is absorbed by tumor cells at a much higher rate, which can then be detected on imaging. The exact explanation for this effect has been debated, but one possibility is that avoiding oxidative phosphorylation evades potential pathways and breakdown products that may result in mitochondrial stress that could halt cell division (13). Furthermore, incomplete breakdown of products such as glucose allows for rapid generation of cellular building blocks that are needed for proliferation. Complete oxidative phosphorylation, by contrast, results in breakdown of the energy source into carbon dioxide, which does not contribute to the biomass of the tumor. By relying on aerobic glycolysis, these cells can continue to divide rapidly and increase in biomass (15).

## Warburg Effect in Prostate Cancer

While a large number of solid tumor cells adhere to this Warburg effect, prostate cancer has a markedly different phenotype. As noted above, benign prostate cells evade oxidative phosphorylation at baseline. It has been shown that early prostate cancers rely on lipids and other energetic molecules for energy production and not on aerobic respiration (16, 17). Therefore, the Warburg effect does not hold consistent in the pathogenesis of prostate cancer, as these cells do not have the increased glucose uptake (18). Clinically this is relevant, as these cancers will not appear on FDG PET scans. It is only in the late stage with numerous mutation events that prostate cancer will begin exhibiting the Warburg effect and have a high glucose uptake. The exact nature of bioenergetics in early prostate cancer cells is still being worked out, and work is being done on specific pathways as discussed below.

## Prostate Cancer Metabolic Changes

### Citrate/Zinc

As noted above, the hallmark of the healthy prostate epithelial cell is the zinc accumulating, citrate synthesizing phenotype. However, a well-noted shift occurs within malignant prostate cells. Prostate cancer cells reverse this phenotype and adopt a zinc wasting, citrate oxidizing phenotype, thereby representing a major shift in energy metabolism (19). This shift allows these cells to utilize the Krebs cycle and subsequent oxidative phosphorylation (Figure 1). It has long been identified that prostate cancer does not conform to the standard Warburg effect seen in most cancers. Unlike most cancer cells that resort to aerobic glycolysis, prostate cancer cells exhibit a higher level of citric acid cycle activity compared to benign cells (10).

Accumulation of zinc may also lead to a mitochondrial apoptotic phenotype within prostate cells. Therefore, malignant cells preferentially decrease the amount of stored zinc in order to avoid cell death (20). This reduction in zinc in malignant cells may be due to alterations in a zinc transporter ZIP1 (19). Altering these transporters does not allow the concentration of zinc to reach levels sufficient to inhibit *m-aconitase*. Thus, it has been argued that the altered zinc and citrate phenotype in prostate cancer has a dual role. Not only does the return to citrate oxidization increase the energy available for cell growth but also by avoiding an increased concentration of zinc these cells avoid apoptotic regulation (3). Further research is being done to better understand how dietary zinc can influence prostate malignancy as well as how the zinc/citrate metabolic path can be targeted therapeutically.

### Lactate

As noted above, later stage prostate cancer cells do begin utilizing aerobic glycolysis leading to an increased intracellular concentration of lactic acid. This build up of lactic acid is toxic so to compensate, cancer cells express monocarboxylate transporters (MCTs) to reduce intracellular lactate levels (Figure 1). Prostate cancer cells have shown to be heterogenic in the level of MCT expression between patients. A more avidly MCT-producing phenotype has been correlated with a more aggressive cancer and worse prognosis (21). By blocking MCT activity, cells may accumulate toxic metabolic products at a faster rate (21). Thus, the expression of lactate shuttles within prostate cancer represents a potential future target as well as a potential diagnostic and prognostic indicator (22).

### Amino Acid Metabolism

As the building blocks of proteins and key intermediates, amino acids and their metabolism have been of an increasing interest in the field of cancer. Differential utilization of particular amino acids has been observed for promotion of cancer cell growth. Furthermore, the specific utilization of amino acids by tumor cells may be useful in determining the aggressiveness of the disease (23). Research into the amino acids glutamine and arginine has become an active area in the field of prostate cancer.

### Glutamine

Glutamine is involved in numerous energetic pathways within cells. A key pathway within benign cells is the transformation into glutamate by glutaminase. This glutamate can transform into alpha-ketoglutarate that is then shuttled to the Krebs cycle (Figure 1).

Glutamine uptake and use has been shown to be elevated in multiple cancers including prostate cancer primarily for *de novo* lipid synthesis, which can contribute to such processes as building cell walls. Under hypoxic conditions commonly seen in tumors, reductive glutamine metabolism by the protein isocitrate dehydrogenase 1 has been shown to promote lipid formation. However, this mechanism is not fully understood (24). Lipids are a key fuel source of prostate cancer cells, particularly early cancer cells.

Glutaminolysis is a mechanism that cancer cells utilize to produce ATP. In prostate cancer cells, glutaminolysis is predominantly performed by the enzyme glutaminase-1. Upregulation of glutaminase-1 has been shown within prostate cancer (25, 26). Therefore, inhibiting the action of glutaminase-1 may theoretically dysregulate the glutamine-based energy production of prostate cancer cells. Indeed, an *in vitro* study has shown that inhibiting glutamine uptake limits the proliferation and invasiveness of prostate cancer (27). As such, interest has risen in the possibility of blocking glutamine uptake clinically.

### Arginine

Arginine is a non-essential amino acid that has been shown to have a variety of roles in the growth of normal cells. As an amino acid, it has an important role in the synthesis of proteins (28). Arginine can be converted to both proline and glutamine. It also has a unique function in the generation of nitric oxide (NO), a compound with a wide array of cellular effects. NO is believed to have an important role in various processes within cancerous cells; however, the exact role of NO in cancer is not well understood at this time (28). A variety of studies have shown that arginine plays an important role in the maintenance of a malignant phenotype in a variety of cancers, including prostate cancer.

While the exact impact that arginine expression has on cancer cells is not well understood, studies have shown that highly available supply of arginine is needed to continue the growth of prostate cancer (29). As such, it has become a potential therapeutic target as deprivation has been shown to lead to death of cancerous cells in multiple studies (28). Arginine can be synthesized from ornithine, a key component of the urea cycle. Ornithine and carbamoyl phosphate are catalyzed by ornithine carbamoyl transferase into citrulline. Citrulline is subsequently transformed into arginine by arginosuccinate synthase. *In vitro*, common prostate cancer cell lines have shown to produce a lower amount of ornithine carbamoyl transferase (28). They have subsequently been shown to be sensitive to treatment by recombinant human arginase (30). Arginine deiminase has also become a popular approach for treatment for prostate cancer, and *in vitro* studies have shown that arginine deiminase can kill susceptible cancer cells by starving the cells of arginine (31). A combination of arginine deprivation with arginine deiminase is being studied as combo therapy in cancer patients in Phase II clinical trials (32).

### Lipid

Many cancers consume lipids in order to produce energy and avoid the citric acid cycle. Prostate cancer cells often utilize lipids derived from androgens through the expression of an androgen receptor (33). However, these cells can also utilize *de novo* lipid synthesis to produce fatty acids in order to obtain energy. This shift to a lipid-producing phenotype is a key turning point in the progression of prostate cancer. The *de novo* lipid producers have ability to produce the key energetic molecules for growth without the regulation of androgens (Figure 1). Clinically, this is problematic as it represents a disease that is unresponsive to androgen deprivation therapy, known as castration-resistant prostate cancer (34).

Research has shown that certain prostate cancer cells overexpress certain markers that are key in the ability to produce *de novo* lipids (35). These include fatty acid synthase (FASN), sterol regulatory element binding protein 1 (SREBP1), and steroyl CoA desaturase among others. Steroyl CoA desaturase is a key enzyme in the formation of monounsaturated fatty acids from larger saturated fatty acids. In some animal models, steroyl CoA desaturase regulation has shown potential as a therapeutic target to inhibit the progression of prostate cancer (35).

### FASN

The enzyme FASN functions to help synthesize long-chain fatty acids. It has long been accepted that a common phenotype within prostate cancer is the upregulation of FASN activity and is of clinical utility as a pathologic biomarker that is occasionally examined in a special stain. Furthermore, there appears to be a dysregulation of FASN where it no longer needs the external stimulation to activate. SREBP1 is stimulated by androgen signaling and epidermal growth factor and has been found to be overexpressed in prostate cancer, leading to the upregulation of FASN (36).

It is believed by some that unregulated FASN activity within prostate tissue is itself the beginning of malignant phenotype and has been argued to be necessary for prostate cancer growth maintenance (37). The use of lipid by the prostate cancer cells illustrates the anabolic pathways that these cells utilize in order to maintain energy and growth and bypass potential degenerative pathways.

### Phosphatase and Tensin Homolog (PTEN)

Another element in metabolism of prostate cancer is PTEN is a tumor suppressor whose activation is of importance as it inhibits the activity of protein kinase B (PKB). The PKB signaling pathway promotes cell survival and proliferation as well as migration (38). It is of importance in signaling cells to progress toward apoptosis as well as arresting the cell cycle. PTEN has been shown to promote an anti-Warburg effect by promoting oxidative phosphorylation and downregulating glycolysis. Downregulation of PTEN has been correlated to a more severe progression and metastasis of prostate cancer (39).

### Glucose

The exact role of glucose metabolism and prostate cancer has not been well defined; however, it does appear to have involvement in the progression of the disease and cellular division (40). Further proof of this involvement is a correlation between individuals with diabetes mellitus and increased severity of cancer phenotype (41). While glucose uptake does not appear to be increased early in prostate cancer cells as mentioned above, there appears to be an important relationship between these factors, particularly in late stage disease (42).

### Androgen

It has been long understood that androgen receptor signaling leads to an increase in the growth of prostate cancer cells. Androgen signals are needed in order to promote *de novo* lipid synthesis, which is required for the growth and survival of these tumor cells (43). Androgen suppression therapy has been used clinically to prevent progression of disease. With prolonged treatment, cells may develop resistance to this treatment. Recently, prostate cancer cells have been shown to have the ability to self-produce steroids, a proposed path by which cells resist androgen deprivation treatment. Prostate cancers that are resistant to androgen deprivation therapy progress to have a poor prognosis, and further treatment options are limited (44).

### AMP Protein Kinase (AMPK)

5′ AMPK is a major site of convergence for many metabolic pathways within the cell and acts as a key energy sensor. Modulation of AMPK therefore leads to numerous alterations within cellular metabolic profiles. Within prostate cancer cells, multiple downstream effects from androgen signaling have been shown to act through AMPK activity (45). As the impact of androgen to prostate cancer has been well established, this has become an area of therapeutic interest. Recently, *in vitro* studies have shown that altering the metabolic activity of AMPK has shown to reduce the prostate cancer growth (44, 46).

### Metabolomics

Metabolomics of prostate cancer is an emerging and active area of research. Metabolomic analyses can theoretically be used to further refine the current understanding of prostate cancer cell metabolic activity by determining the concentration of the excreted products. As previously noted, the long used standard of PSA testing has come under increasing scrutiny recently. Better understanding of metabolomics profile can therefore have an immense benefit in potentially determining new screening and risk stratification methods both for initial diagnostic and follow-up care (47). Advancements in metabolomics technologies have further spurred interest in this field as the use of gas chromatography and mass spectroscopy and NMR spectroscopy has now been utilized in profiling prostate cancer products. Research has been conducted in using a urine sample to assess metabolic profiles (8). If efficacious, this would represent a less interventional testing modality for patients. However, the results from this method have been mixed and variability in urine collection represents a barrier to performance. Overall, metabolomics represents an exciting avenue for discovery as the presence of certain metabolites may underscore the importance of highly utilized pathways, and help determine future care (5, 8, 47–50).

## Conclusion

As the leading form of cancer among men, prostate cancer represents a major burden of disease within the United States. An active area of research is in the metabolism of prostate cancer. There are a variety of metabolic alterations that occur when a benign cell becomes malignant. There is still a great deal of research to be done, as many of the mechanisms of cellular cancer metabolisms are not well understood. With better knowledge comes the possibility of creating better treatment options and diagnostic testing.

## Author Contributions

The authors were involved with the oversight and revision of the manuscript.

## Conflict of Interest Statement

The authors declare that the research was conducted in the absence of any commercial or financial relationships that could be construed as a potential conflict of interest.
